# A Virtual Introduction to Mental Health Tribunals: A Pilot Project

**DOI:** 10.1192/bjo.2022.127

**Published:** 2022-06-20

**Authors:** Razan Halawa, Seona Duroux

**Affiliations:** Avon and Wiltshire Mental Health Partnership NHS Trust, Bristol, United Kingdom

## Abstract

**Aims:**

This training aimed to familiarise the trainee of the mental health tribunal process and its delivery in the virtual form, improve the trainee's ability to provide targeted and succinct evidence both in written and verbal form.

**Methods:**

This project followed a PDSA cycle. We targeted core trainees in their 3rd year (CT3s) for this pilot for practical reasons, we've also asked one third year nursing student to participate. We have started with a pre-intervention survey to obtain knowledge and confidence levels with regard to tribunals and identify further training needs. We used one of the slots available on Thursday MRCPsych course schedule to conduct the pilot. We have identified several people who are willing to participate in the teaching process, including an inpatient unit consultant, a judge, an experienced panel psychiatrist.

We have obtained teaching material from the MHA office at the trust. On the day of the pilot, we prepared introductory material in the form of power point presentation about tribunals and how to write reports. We then introduced our virtual patient “Mike” whom we based the report writing workshop and MOCK tribunal on using theoretical nursing and doctor entries. The teaching was followed by post intervention qualitative feedback. Data of pre and post intervention were moved to an excel spreadsheet for further analysis. We will take the results from this pilot to inform the next cycle of the project.

**Results:**

Quantitative data: The training module was conducted over Zoom. Pre and post intervention surveys indicated an improvement in the trainees’ knowledge and confidence scores of 28–44% as described in figure 1.

Qualitative data: The trainees and facilitators provided very positive qualitative feedback. Themes mentioned were related to comprehensiveness of training material, confidence gained in providing evidence, range of information covered, on hand experience, experts presence, relevance for multidisciplinary training cohort. Areas of improvement included minor technical difficulties, suggestion of more time spent on nature and degree, and involving service user and lay person representatives.

**Conclusion:**

The above analysis and feedback suggests a successful run of the first pilot. We will aim to increase representation and allow more time for the some of the key learning points like understanding the difference between nature and degree in relation to MHA. We will continue to liaise with the department of Medical Education at Severn Deanery and We will also run the project at a wider scale including more nursing students and trainees of different levels.

